# Gold‐Triggered Uncaging Chemistry in Living Systems

**DOI:** 10.1002/anie.201705609

**Published:** 2017-08-09

**Authors:** Ana M. Pérez‐López, Belén Rubio‐Ruiz, Víctor Sebastián, Lloyd Hamilton, Catherine Adam, Thomas L. Bray, Silvia Irusta, Paul M. Brennan, Guy C. Lloyd‐Jones, Dirk Sieger, Jesús Santamaría, Asier Unciti‐Broceta

**Affiliations:** ^1^ Cancer Research (UK) Edinburgh Centre MRC Institute of Genetics and Molecular Medicine University of Edinburgh UK; ^2^ Department of Chemical Engineering and Environmental Technology and Institute of Nanosciences of Aragon (INA) University of Zaragoza Spain; ^3^ Networking Research Center on Bioengineering, Biomaterials and Nanomedicine, CIBER-BBN 28029 Madrid Spain; ^4^ Centre for Neurogeneration, The Chancellor's Building University of Edinburgh UK; ^5^ Centre for Clinical Brain Sciences University of Edinburgh UK; ^6^ School of Chemistry, King's Buildings, West Mains Road University of Edinburgh UK

**Keywords:** bioorthogonal, fluorescent probes, gold, heterogeneous catalysis, prodrugs

## Abstract

Recent advances in bioorthogonal catalysis are increasing the capacity of researchers to manipulate the fate of molecules in complex biological systems. A bioorthogonal uncaging strategy is presented, which is triggered by heterogeneous gold catalysis and facilitates the activation of a structurally diverse range of therapeutics in cancer cell culture. Furthermore, this solid‐supported catalytic system enabled locally controlled release of a fluorescent dye into the brain of a zebrafish for the first time, offering a novel way to modulate the activity of bioorthogonal reagents in the most fragile and complex organs.

Seminal works showcased the capabilities of foreign transition metal catalysts in mediated chemoselective transformations within cells.^1^ More recently, the emerging field of bioorthogonal catalysis^2^ has produced a wealth of creativity in a variety of applications, ranging from biomolecule labeling,3a–3c metabolite detection3d and intra/subcellular probe release,3e–3h to in situ enzyme3i,3j and prodrug activation.3k–3o Substoichiometric activity and access to a greater diversity of chemical processes and functionalities are some of the added benefits provided by abiotic transition metals to the current bioorthogonal toolbox, thus expanding the boundaries of this central field of research.^4^ One of the latest additions to this area was recently reported by Tanaka and coworkers,^5^ who developed a novel strategy for in vivo protein labeling mediated by glycoalbumin‐bound gold(III) complexes (Scheme 1). Despite recent advances in the field, many challenges lie ahead as transition metal catalysts show limited biocompatibility in living systems in terms of catalytic versatility and inherent toxicity.

**Scheme 1:**
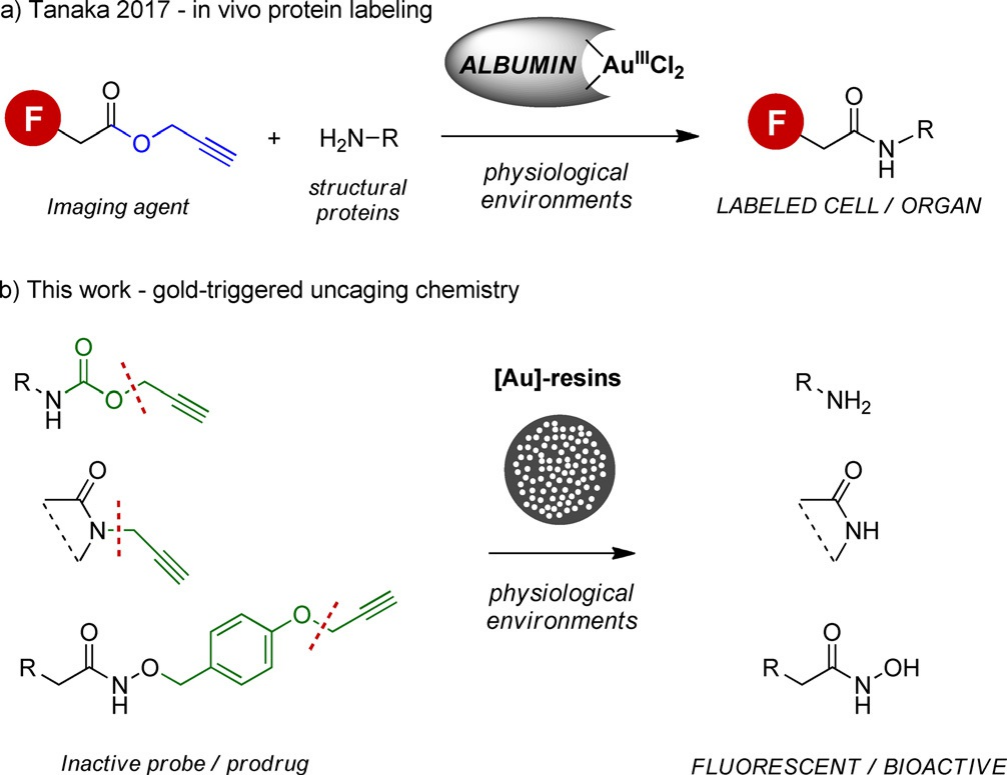
a) Au^III^‐mediated bioorthogonal amidation reported by Tanaka and coworkers.^5^ b) The solid‐supported gold‐catalyzed uncaging strategy developed in this work.

Gold catalysis has received enormous attention in organic synthesis over the last decades.^6^ Among the chemical properties of gold that stand out are its preference to coordinate with alkynes in the presence of other functional groups, including alkenes.^6^, ^7^ Solid‐supported gold nanoparticles (Au‐NP) have also attracted the interest of chemists searching for greener catalysts, because such catalysts are recyclable, gold is safe to handle, and it demonstrates a remarkable ability to catalytically mediate oxidative reactions at or even below ambient temperatures.^7^ In the chemical biology field, however, the chemistry of gold is dominated by near‐covalent S−Au bonding.^8^ This spontaneous bond formation provides the basis for the bottom‐up self‐assembly of monolayers functionalized with a multitude of small molecules and biomolecules at the surface of the metal—a highly reliable process that has found widespread application in nanotechnology, biotechnology, and theranostics.^8^ Because of the high affinity of thiols for gold and their ubiquitous presence in peptides and proteins, the attractive alkynophile properties of Au‐NP pass unnoticed in the biological milieu. We envisioned that embedding Au‐NP in a solid support would serve to protect the metal nanostructures from large thiol‐rich biomolecules, while allowing alkyne‐functionalized small molecules to enter and undergo gold‐mediated chemistry even in biological systems. Importantly, based on the high biocompatibility of metallic gold, such a device would be optimal to catalyze bioorthogonal transformations in vivo. In a suitable shape and size, this functional device could potentially be implanted by a surgeon at the anatomical site of a localized cancer (for example, the brain) and enable the local “manufacture” of different drugs—in a catalytic fashion—from systemically administered innocuous starting materials. This unique delivery method would offer the benefits of drug release systems^9^ (that is, focal treatment and reduced general side effects) with fewer limitations (for example, extended lifetime and access to multiple therapies).

Towards this goal, herein we report the first example of bond‐cleavage chemistry mediated by heterogeneous gold catalysts in living systems (Scheme 1)—a previously overlooked chemical reactivity of gold that facilitates the efficient bioorthogonal uncaging of various clinically approved anticancer drugs in cancer cell culture and the first intracranial activation of a bioorthogonal probe in zebrafish.

Solid‐supported gold catalysts (Figure 1 a,b) were prepared by in situ generation of Au‐NP within a polyethylene glycol (PEG)‐grafted low‐cross‐linked polystyrene matrix. In short, amino‐functionalized TentaGel^®^ HL resins of 75 microns in diameter (Rapp Polymere GmbH) were treated with tetrachloroauric(III) acid and sodium hydroxide in water:tetrahydrofuran (THF) followed by reduction with tetrakis(hydroxymethyl)phosphonium chloride (THPC; see full synthesis in the Supporting Information). THPC was used to control particle growth and distribution because of its relatively mild reducing properties.^10^ High‐angle annular dark‐field (HAADF) scanning transmission electron microscopy (STEM) images of ultramicrotome cross‐sections of the resins showed uniformly dispersed polyhedral nanocrystals of 30 nm average diameter across the polymer framework (Figure 1 b; Supporting Information, Figure S1). X‐ray photoelectron spectroscopy (XPS, Supporting Information) analysis detected an incremental gradient concentration of gold from the surface to the core of the resins, with an Au^0^/Au^δ+^ ratio ranging from 7 (periphery) to 19 (interior).

**Figure 1:**
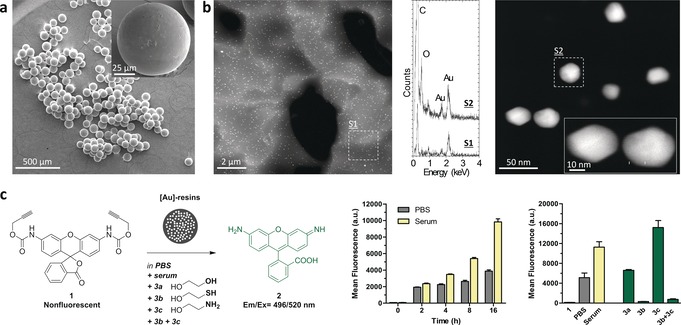
a) Scanning electron microscopy (SEM) images of [Au]‐resins. b) HAADF‐STEM images of a cross‐section of a [Au]‐resin at different magnifications and energy‐dispersive X‐ray (EDX) spectra of highlighted areas. c) Cleavage of *N*‐Poc‐protected prodye **1** (20 μm) in the presence of [Au]‐resins (1 mg mL^−1^) at physiological conditions (pH 7.4, 37 °C). Central panel: reaction kinetics in PBS or serum. Right panel: fluorescence analysis at 24 h in different conditions. Negative control: reagent **1** without [Au]‐resins.

To investigate the properties of the [Au]‐resins in physiological conditions, the devices were incubated with nonfluorescent compound **1**, which releases strongly fluorescent Rhodamine 110 (**2**) upon *O*‐depropargylation (Figure 1 c). Reactions were performed in phosphate buffered saline (PBS; pH 7.4) at 37 °C, either with or without serum, and fluorescence was measured with a spectrophotometer. Analysis revealed the rapid generation of fluorescence under both conditions, with the presence of serum achieving a much greater fluorogenic effect (ca. 25 % yield), demonstrating the feasibility of performing Au‐NP‐mediated chemistry beyond the S−Au bonding in biocompatible environments. Time‐course and reusability studies (Supporting Information, Figures S3 and S4) provided proof of the durability of the devices and their capacity to activate multiple doses of masked reagent—although a gradual decay in activity was observed over time.

To better understand the enhanced catalytic activity of the [Au]‐resins in the presence of serum, we investigated the influence of OH, SH, and NH_2_ groups (nucleophilic groups found in serum proteins) in the conversion of **1** into **2** by adding excess ethylene glycol (**3 a**), 2‐mercaptoethanol (**3 b**), and ethanolamine (**3 c**), respectively. These low molecular weight chemicals were used to facilitate diffusion throughout the resins, thereby maximizing their interaction with internal Au‐NP. All the reactions were carried out in PBS at 37 °C. Although **3 a** had a relatively minor effect on the catalytic capacity of the devices, substantial variations in activity were observed in the presence of **3 b** and **3 c** (Figure 1 c). Thiol **3 b** suppressed the reactivity of the resins almost completely, whereas addition of amine **3 c** boosted catalytic activity significantly. The combined presence of an excess of both **3 b** and **3 c** resulted in low levels of fluorescence intensity. This prompted us to investigate the influence of glutathione in the reaction, a natural reductant that contains a SH and an NH_2_ group in its structure. Since glutathione is found ubiquitously in the human plasma and the interstitial fluid at a concentration of 2–20 μm,^11^ we studied the reaction of **1** and [Au]‐resins at concentrations ranging from 10 to 400 μm. As shown in the Supporting Information, Figure S5, increasing glutathione levels up to 50 μm promoted the reaction, whereas greater concentrations led to a substantial reduction in fluorescence intensity. Notably, a large regain in catalytic activity was achieved upon addition of an extra milligram of [Au]‐resins to the inhibited reactions. In contrast, if the concentration of probe **1** was augmented, no significant increase in fluorescence generation was observed. These results indicate that S−Au bonding of glutathione molecules on the surface of the Au‐NP promote the dealkylation reaction until a saturation threshold is reached (see rationale in Scheme 2 a). Over the saturation limit, gold‐bound biomolecules will coat most active sites on the Au‐NP surface, thus hindering gold–alkyne coordination. A series of tests carried out to monitor and analyze the reaction (Supporting Information, Figures S6 and S7) corroborated that Rhodamine **2** was the main product of the reaction, along with intermediates that could correspond to organogold species. However, no reaction byproducts were isolated or identified, which points to the production of short‐lived compounds. Based on these experimental observations, we tentatively propose a dealkylation pathway whereby gold acts as a π‐acid to activate the nucleophilic addition of biomolecules onto the terminal alkyne group, leading to release of the leaving group (for example, a dye or drug) and the generation of reactive allenyl byproducts (Scheme 2 b) that isomerize or hydrolyze under the reaction conditions.

**Scheme 2:**
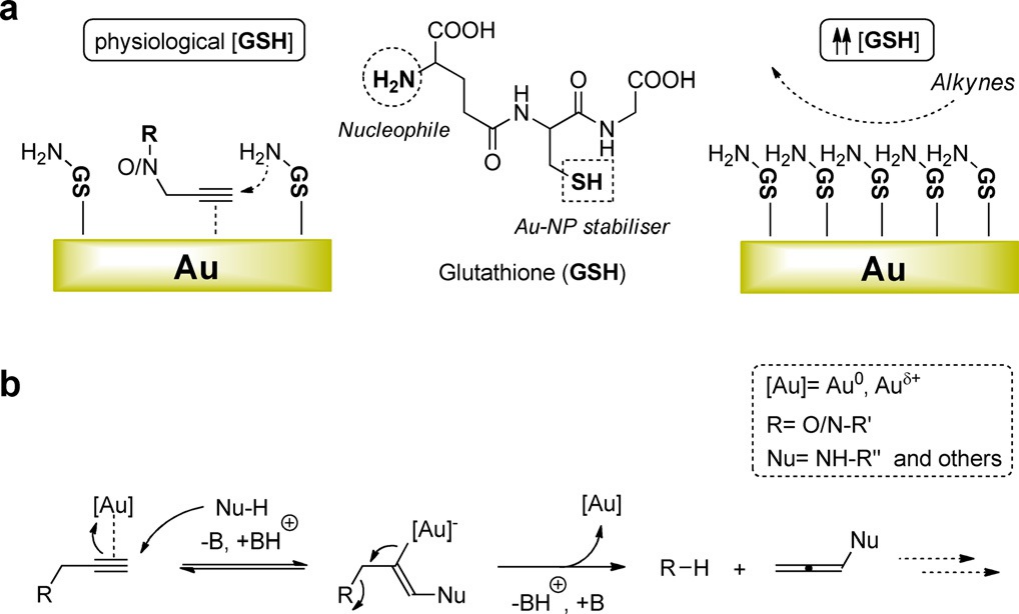
a) Rationale for the assisting–inhibiting roles of glutathione in [Au]‐catalyzed *O*/*N*‐propargyl cleavage reactions. b) Tentative reaction mechanism for the [Au]‐triggered depropargylation of **1** and **4 a**–**c** in the biological milieu.

Prior to testing the catalytic properties of the devices in cell culture, viability assays (PrestoBlue® reagent) were performed to determine the tolerance of cells to the presence of solid‐supported gold. As anticipated, [Au]‐resins were found to be fully biocompatible at the concentrations tested (Supporting Information, Figure S8).

Subsequently, the bioorthogonal [Au]‐triggered release of a structurally diverse selection of clinically used anticancer drugs was investigated in culture with human lung cancer A549 cells. Three different drug precursors were tested (see Figure 2 a): Pro‐FUdR ^[12a]^ (**4 a**), POB‐SAHA12b (**4 b**), and *N*‐Poc‐DOX (**4 c**; a novel drug precursor inspired by prior designs3m, 13). Cells were treated with **4 a**–**c** and [Au]‐resins separately (negative controls) or in combination (activation assay), and unmodified drugs **5 a**–**c** used as the positive controls. Remarkably, while prodrugs **4 a**–**c** did not elicit any effect on their own, potent anticancer activity was displayed in combination with [Au]‐resins (Figure 2 a); unequivocal evidence that the active drugs are released in situ by heterogeneous gold chemistry. Reuse of the [Au]‐resins in three consecutive prodrug activation cycles confirmed the capacity of the devices to activate multiple drug doses (Supporting Information, Figure S9). The synthesis of drugs **5 a**–**c** was also verified in vitro (Supporting Information, Figure S10), confirming the capability of gold to cleave both *O*‐ and *N*‐propargyl groups from a range of molecules based on structurally different scaffolds. These studies support a potential application scenario where gold‐functionalized implants could be used in situ to modulate the spatiotemporal generation of chemotherapeutics from inactive precursors in the treatment of localized malignancies such as brain or prostate cancer.

**Figure 2:**
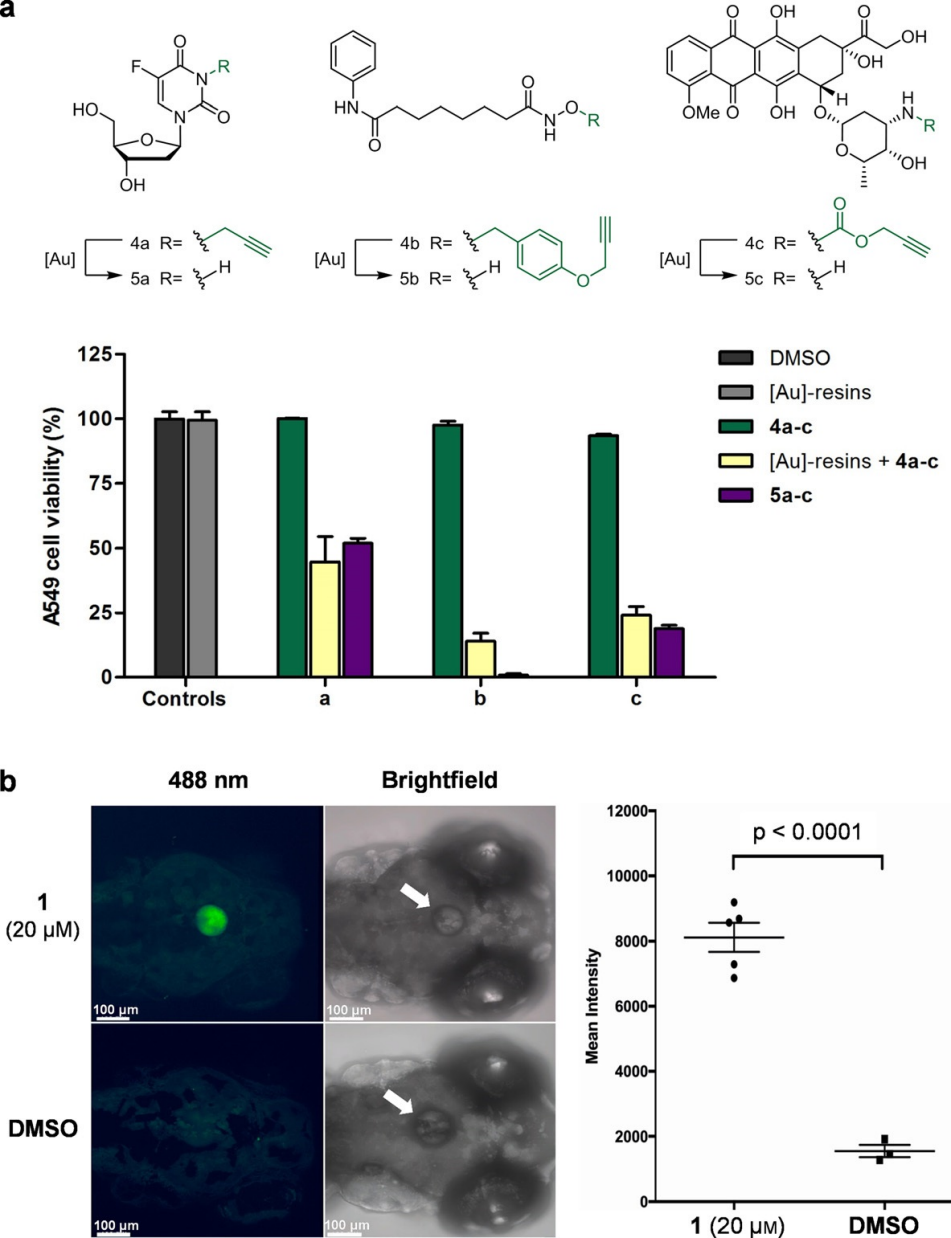
a) Gold‐triggered activation of prodrugs **4 a**–**c** in A549 cancer cell culture. Negative controls: [Au]‐resins (1 mg mL^−1^); **4 a**–**c** (10, 100, and 1 μm, respectively). Positive control: **5 a**–**c** (10, 100, and 1 μm, respectively). Prodrug activation assay: [Au]‐resins+**4 a**–**c** (10, 100, and 1 μm, respectively). Cell viability was measured at day 4 using PrestoBlue reagent. Error bars: ±SD from *n=*3. b) Bioorthogonal gold‐mediated release of green fluorescent Rhodamine 110 from precursor **1** in the brain of a zebrafish. The presence of the [Au]‐resin is indicated with a white arrow. Study of fluorescence intensity shows high statistical significance compared to the negative control (DMSO).

Encouraged by the biocompatibility and catalytic properties of the [Au]‐resins, we embarked on an innovative study to evaluate the capacity of the devices to convert nonfluorescent **1** into Rhodamine **2** inside the cranium of zebrafish embryos. Firstly, a single [Au]‐resin was carefully transplanted into the optic tectum, a small anatomical cavity^14^ of the brain of zebrafish larvae. After the surgery, either reagent **1** (activation assay) or just dimethyl sulfoxide (DMSO; negative control) were added to the medium and embryos imaged at 24 h (*n*=5). Because of its lipophilicity, prodye **1** can enter the zebrafish through the skin and/or by ingestion and distribute systemically, but will only be converted into fluorescent compound **2** upon reaction with the [Au]‐bead. As shown in Figure 2 b, a strong green fluorescent signal originating from the [Au]‐resin was observed only when incubated with **1**, confirming the local generation of Rhodamine **2**. Prolongation of the study by three additional days corroborated previous observations regarding the sustained functionality of the devices (Supporting Information, Figure S11). This study, which represents the first bioorthogonal organometallic reaction to be locally performed in the brain of a living animal, indicates that heterogeneous gold catalysts have the capacity to mediate in vivo bioorthogonal release of functional reagents in a spatially controlled manner.

In conclusion, we have developed a heterogeneous catalytic system that enables access to chemical properties of Au‐NP that were previously out of our reach in biological environments. Such devices triggered the bioorthogonal uncaging of a structurally diverse selection of cytotoxic precursors through an unexplored chemical reactivity of gold, providing a novel and safe method to activate therapeutics by nonbiological chemical stimuli.3k–3o, 13, 15 Furthermore, this solid‐supported catalyst enabled—for the first time—the locally controlled release of a fluorescent dye in the brain of a zebrafish. This notable breakthrough expands our capacity to chemically modulate the activity of bioorthogonal reagents in the most fragile and complex organs.

## Conflict of interest

The authors declare that compounds **4 a** and **4 b** are protected under patent.

## Supporting information

As a service to our authors and readers, this journal provides supporting information supplied by the authors. Such materials are peer reviewed and may be re‐organized for online delivery, but are not copy‐edited or typeset. Technical support issues arising from supporting information (other than missing files) should be addressed to the authors.

Supplementary
