# Supplementary material for: Gold‐Triggered Uncaging Chemistry in Living Systems
Source: Angew Chem Int Ed Engl. 2017 Aug 9;56(41):12548–52. doi: 10.1002/anie.201705609 (PMC5655737; doi:10.1002/anie.201705609)
Supplement: Supplementary file 1 — Supplementary [file ANIE-56-12548-s001.pdf]

Angewandte Chemie
Eine Zeitschrift der Gesellschaft Deutscher Chemiker

Supporting Information

Gold-Triggered Uncaging Chemistry in Living Systems

Ana M. Pérez-López, Belén Rubio-Ruiz, Víctor Sebastián, Lloyd Hamilton, Catherine Adam, Thomas L. Bray, Silvia Irusta, Paul M. Brennan, Guy C. Lloyd-Jones, Dirk Sieger, Jesús Santamaría, and Asier Unciti-Broceta**

anie_201705609_sm_miscellaneous_information.pdf

Index

1. General S3

2. Synthesis and characterization of [Au]-resins S3

2.1. Synthetic procedure S3

2.2. Characterization S3

3. Synthesis and characterization of 1 and 4a-c S6

3.1. Synthesis of O-(*4-nitrophenyl)-O'-propargylcarbonate* S6

3.2. Synthesis of *N-Poc-DOX* (4c) S6

4. [Au]-catalysed deprotection of 1 S8

4.1. Fluorogenic assay S8

4.2. Reusability study S8

4.3. Time-course study S9

4.4. Study of the influence of glutathione S10

5. Tests performed to identify reaction products and intermediates S11

5.1. Reaction of 1 with [Au]-resins and benzylamine. TLC-MS analysis S11

5.2. Reaction of 6 with [Au]-resins and 3c in DCM. LCMS study S12

6. Biological studies S13

6.1. Cell culture S13

6.2. Cell viability studies S13

6.3. [Au]-mediated activation of 4a-c in cell culture S13

6.4. Multidose prodrug activation study in cancer cell culture S14

7. In vitro prodrug deprotection studies S15

8. [Au]-resin implantation in zebrafish brain and *gold*-mediated activation of 1 S16

9. References S18

1. General

Chemicals and solvents were purchased from Fisher Scientific, Sigma-Aldrich or VWR International Ltd. Resins were purchased from Rapp Polymere GmbH. Drugs were purchased from Cayman Chemical Company (vorinostat), MedChem Express (doxorubicin) and LKT Laboratories (floxuridine).

NMR spectra were recorded at ambient temperature on a 500 MHz Bruker Avance III spectrometer. Chemical shifts are reported in parts per million (ppm) relative to the solvent peak.

All compounds used in the biological experiments were > 95% pure, as measured by HPLC. Stock solutions (100 mM) were prepared in biological grade DMSO.

2. Synthesis and characterization of [Au]-resins.

2.1. Synthetic procedure. TentaGel® HL NH_2 resins (250 mg, 0.4-0.6 mmol / g, particle size 75 μm) were added into a 25 mL Biotage microwave vial and suspended in THF (2.5 mL). A solution of gold(III) chloride hydrate (120 mg, 0.35 mmol) in distilled water (500 μL) was basified with a 1 M NaOH aqueous solution (11 μL). This freshly prepared solution was immediately added to the suspended resins and heated to 60 °C under stirring for 10 min. The mixture was then stirred at r.t. for additional 2 h. Subsequently, the solvents were filtered and the resins washed with DMF (3 × 10 mL), DCM (3 × 10 mL) and methanol (3 × 10 mL). Tetrakis(hydroxymethyl)phosphonium chloride (THPC) solution 80% in water (93 μL) was diluted in distilled water (6 mL) and a 1 M NaOH aqueous solution (11 μL) added. This solution was added to the gold(III)-treated resins and bubbled with a N_2 flow at r.t. for 25 min. The solvents were then filtered off and the resins washed with methanol (3 × 10 mL) and DCM (3 × 10 mL). Resins were then added to a solution of Fmoc-Glu(OH)-OH (64 mg, 0.17 mmol), oxyma (50 mg, 0.35 mmol), *N*,*N′*-diisopropylcarbodiimide (54 μL, 0.35 mmol) and DCM/DMF (3:1, 8 mL) and stirred for 2 h at r.t. The solvents were filtered off and the resins washed with DMF (1 × 10 mL), DCM (3 × 10 mL) and methanol (3 × 10 mL). Finally, resins were dispersed and shaken in a solution of acetic anhydride (60 μL) in DCM (10 mL) for 1h at r.t. The solvents were filtered and the resins were washed with DCM (3 × 10 mL) and methanol (3 × 10 mL). Resins were treated on the wheel overnight with methanol. The solvents were then filtered and resins were dried in an oven at 40 °C for 1 d.

2.2. Characterization.

Electron microscopy analyses. To prepare the samples, [Au]-resins were infiltrated and embedded in a liquid epoxy resin. After curing the resin block, it was sectioned with an Ultramicrotome (Leica EM UC7) equipped with a diamond knife. Sections of 50-70 nm thickness were collected on a copper grid (formvar-200 mesh) coated with carbon film, and allowed to dry in air. Morphological analysis of [Au]-resins was carried out by Scanning Electron Microscopy (SEM, Inspect F50; FEI, Eindhoven, the Netherlands) at the LMA-INA-Universidad Zaragoza facilities

operated at 10–15 kV. This microscope is equipped with different SEM detectors that enable to: 1) Obtain SEM images with secondary electrons (Everhart-Thornley Detector); 2) Obtain SEM images and composition by using a Back Scattering Electron Detector; 3) Elemental chemical analysis by energy-dispersive X-ray microanalysis (EDS). Transmission Electron Microscopy observations were carried out at the LMA-INA-UNIZAR facilities using a FEI Tecnai F30 with a Field Emission Gun operating at 300kV. The microscope SuperTwin® lens allows a point resolution of 1.9 Å. The microscope is fitted with a High-Angle Annular Dark Field (HAADF) detector to operate in STEM mode with Z-contrast imaging.

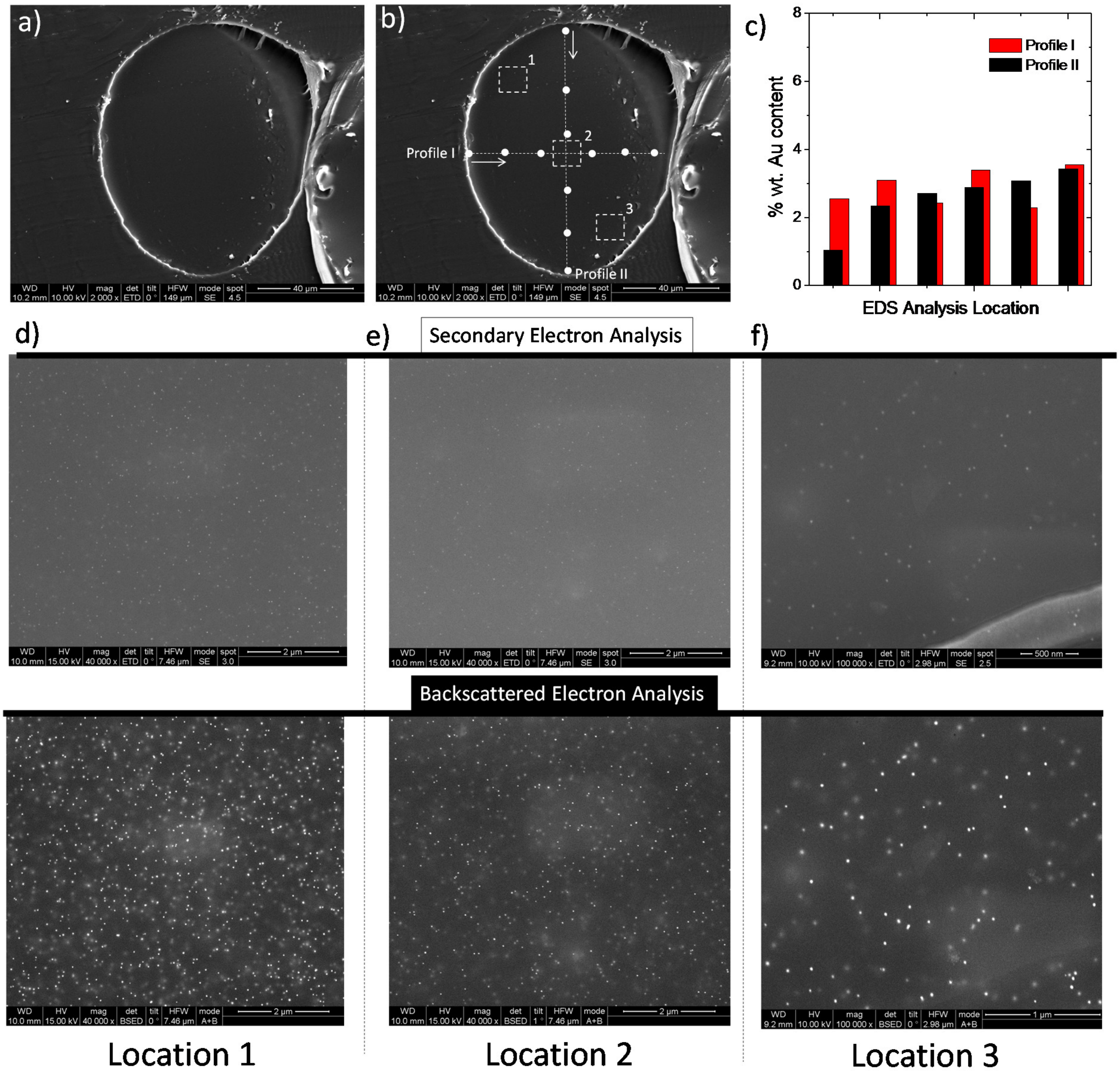

Suppl. Fig. 1. a) SEM cross section image of a [Au]-resin. b) SEM image of a) marked with the locations where energy-dispersive X-ray microanalysis were performed (Profile I and II). c) EDS analysis plot of profiles I and II showing homogenous loading of Au-NP within the resin. d-f) Secondary Electron and Backscattered Electron SEM images of marked areas 1-3 in panel b). Location and homogenous distribution of Au-NP are observed throughout a cross-section of a [Au]-resin.

X-ray photoelectron spectroscopy (XPS) analysis was performed with an AXIS Supra (Kratos Tech.). The spectra were excited by the monochromatized AlKα source (1486.6 eV) run at 15 kV and 15 mA. For the individual peak regions, a pass energy of 20 eV was used. Peak analyses were performed with the CasaXPS software, using a weighted sum of Lorentzian and Gaussian components curves after Shirley background subtraction. The binding energies were referenced to the internal C 1s (284.9 eV) standard. Material composition at different depths was obtained by measuring after sputtering the surface of the sample for a certain time using a gas cluster ion source. Argon ion clusters of mean size 1000 atoms and energy 10kV were used for etching.

Sample	Etching time (s)	B. E. (eV) Atomic %		
		O 1s	C 1s	Au 4f
[Au]-resins	0	533.0<br>15.64%	284.9<br>84.11%	84.2<br>0.25%
	50	533.2<br>17.40%	284.9<br>81.79%	84.3<br>0.81%
	100	533.0<br>17.08%	284.9<br>82.08%	84.2<br>0.84%
	150	533.1<br>16.22%	284.9<br>82.89%	84.2<br>0.89%
	200	532.9<br>15.83%	284.9<br>83.24%	84.1<br>0.93%

Au 4f 7/2

Sample	Etching time (s)	B. E. (eV) Atomic %	
		Au^0	$Au^{\delta+}$
[Au]-resins	0	83.7<br>88%	84.3<br>12%
	50	83.7<br>94%	84.6<br>6%
	100	83.6<br>95%	84.7<br>5%
	150	83.7<br>93%	84.7<br>7%
	200	83.5<br>90%	84.6<br>10%

As shown in the above table, the major gold oxidation state found in the resins was Au^0, while a relatively small proportion consisted of $Au^{\delta+}$ species. Since debate still exists over the oxidation state/s responsible for the catalytic properties of solid supported Au-NP [1], the potential contribution of positively charged / polarized Au species to the catalysis cannot be ignored.

3. Synthesis and characterization of 1 and 4a-d

Prodye (**1**), Pro-FUdR (**4a**) and POB-SAHA (**4b**) were synthesised as previously reported [2-4].

3.1. Synthesis of O-(4-nitrophenyl)-O'-propargylcarbonate. A solution of 4-nitrophenyl chloroformate (0.48 g, 2.4 mmol) in dry DCM (8 mL) was added drop wise to a solution of propargyl alcohol (1.3 g, 2.2 mmol) and pyridine (0.19 mL, 2.4 mmol) in DCM (8 mL) at 0 °C under nitrogen in the dark. The mixture was stirred from 0 °C to r.t. overnight with TLC monitoring for 20 h until full consumption of the alcohol. After concentrating *in vacuo,* the crude residue was re-dissolved in ethyl acetate (70 mL), washed with water (2 x 50 mL) and brine (2 x 50 mL), dried over $MgSO_4$ anhydrous and concentrated *in vacuo,* and the crude purified by flash chromatography (50 % DCM in hexane) to give white crystals (0.31 g, 1.40 mmol, 64 % yield); R_f 0.29 (50 % DCM in hexane). ^1H NMR (500 MHz, $CDCl_3$) δ 8.32 – 8.25 (m, 2H), 7.44 – 7.38 (m, 2H), 4.88 (d, *J* = 2.5, 2H), 2.62 (t, *J* = 2.5, 1H). ^{13}C NMR (126 MHz, $CDCl_3$) δ 155.45, 152.13, 145.74, 125.52, 121.87, 77.37, 76.88, 56.63. HRMS (m/z): $[M+Na]^+$ calcd. for $C_{10}H_7O_5N_1$ $[M+Na]^+$: 244.0216, found: 244.0206.

3.2. Synthesis of N-Poc-DOX (4d). A solution of O-(4-nitrophenyl)-O'-propargylcarbonate (9 mg, 40.9 µmol) in anhydrous DMF (2 mL) was flushed with nitrogen for 10 min, then syringed into a flask containing a solution of doxorubicin HCl (15 mg, 27.3 µmol) and triethylamine (5.75 µL, 40.9 µmol) in anhydrous DMF under nitrogen and stirred at r.t. The reaction was monitored by TLC (10% methanol in DCM) for 20 h to observe the formation of a product with R_f= 0.88 (10% methanol in DCM). The reaction was then diluted with water (50 mL) and extracted with ethyl acetate (4 x 50 mL). The combined organic extracts were concentrated to ~100mL, then washed successively with saturated $NaHCO_3$ (2 x 50 mL), water (2 x 50 mL) and brine (2 x 50 mL), dried over $MgSO_4$ anhydrous and concentrated *in vacuo* with the water bath kept below 40°C, and the crude purified via flash chromatography (0 → 2 % Methanol in DCM) to give dark red powder (12.4 mg, 19.8 µmol, 73 % yield); R_f 0.25 (2% Methanol in DCM). ^1H NMR (500 MHz, $CDCl_3$) δ = 13.96 (s, 1H), 13.22 (s, 1H), 8.02 (d, *J* = 7.6, 1H), 7.77 (t, *J* = 8.1, 1H), 7.37 (d, *J* = 8.4, 1H), 5.49 (d, *J* = 3.9, 1H), 5.28 (s, 1H), 5.16 (d, *J* = 8.5, 1H), 4.74 (dd, *J* = 4.8, 1.8, 2H), 4.59 (d, *J* = 2.1, 2H), 4.50 (s, 1H), 4.12 (q, *J* = 6.5, 1H), 4.06 (s, 3H), 3.85 (s, 1H), 3.65 (d, *J* = 6.4, 1H), 3.26 (dd, *J* = 18.8, 1.5, 1H), 3.02 - 2.96 (m, 2H), 2.41 (s, 1H), 2.31 (d, *J* = 14.7, 1H), 2.19 – 2.12 (m, 1H), 1.87 (dd, *J* = 16.9, 6.5, 2H), 1.75 (td, *J* = 13.2, 4.2, 1H), 1.27 (d, *J* = 6.6, 3H). ^{13}C NMR (126 MHz, $CDCl_3$) δ = 213.97, 187.31, 186.90, 161.24, 156.32, 155.83, 154.75, 135.93, 135.69, 133.72, 133.64, 121.08, 120.03, 118.62, 111.81, 111.63, 100.78, 78.23, 76.78, 74.80, 69.83, 69.68, 67.36, 65.69, 56.84, 52.67, 47.26, 35.80, 34.19, 30.32, 16.97. HRMS (m/z): $[M+Na]^+$ calcd for $C_{31}H_{31}O_{13}N_1$ 648.1687 found 648.1677.

^{1}H NMR spectra of 4c

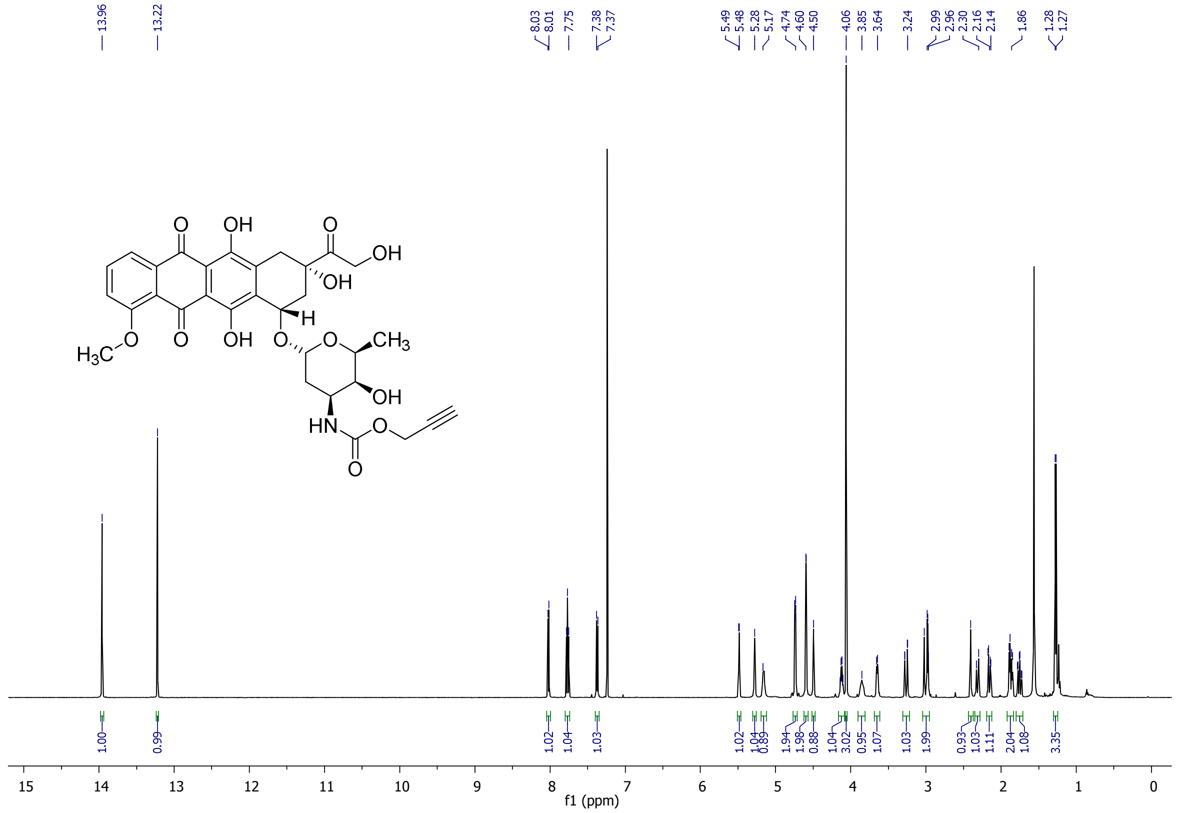

^{13}C NMR spectra of 4c

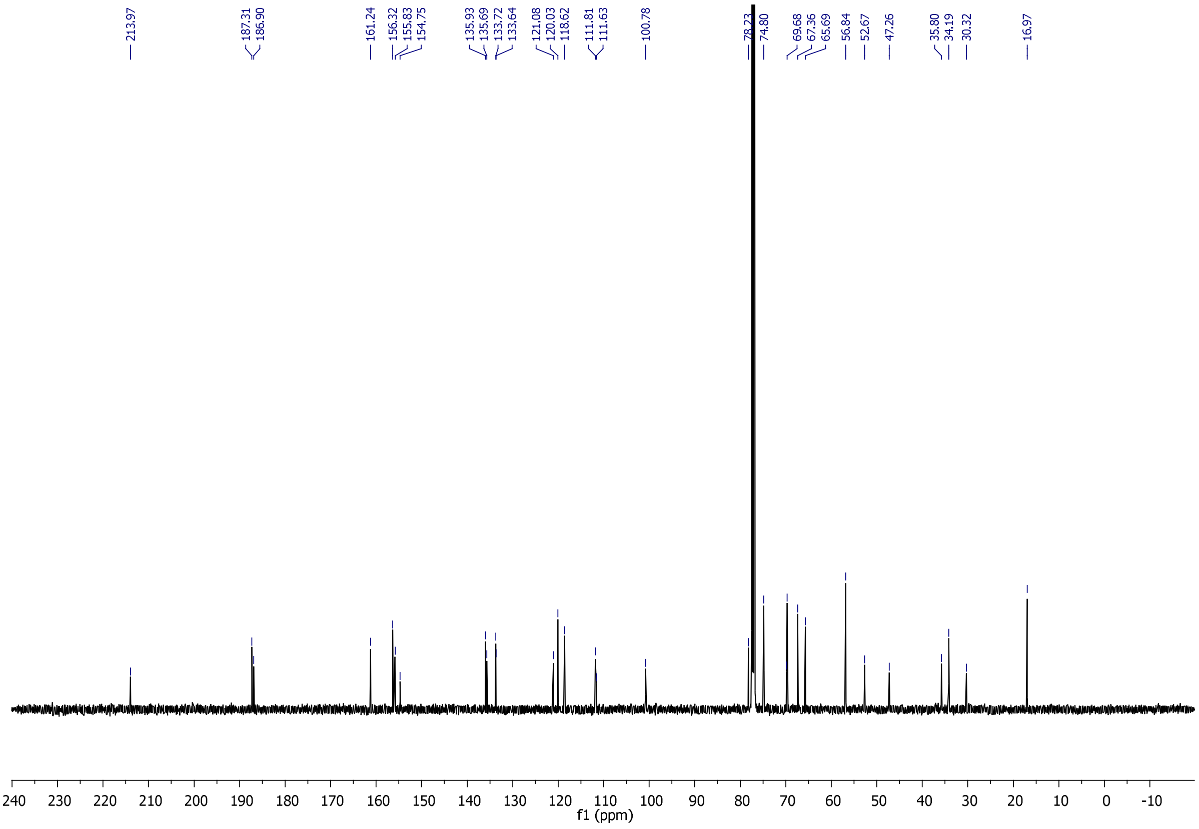

4. [Au]-catalysed deprotection of 1

4.1. Fluorogenic assay. [Au]-resins (1 mg) were added to a 1 mL solution of reagent **1** (20 μM) in PBS (with or without additives). Additives: foetal bovine serum (10 %), compounds **3a** (5 %), **3b** (5 %), **3c** (5 %), or **3b + 3c** (5 % each). The pH of the solutions were adjusted to pH=7.4 with HCl when needed. The mixtures were shaken at 1,200 rpm and 37°C in a Thermomixer and reactions monitored at 2 h, 4 h, 8 h, 16 h (for studies in PBS and serum) and 24 h (for all) by fluorescence in a PerkinElmer Victor multilabel reader (excitation filter at 480nm and emissions filter at 535nm).

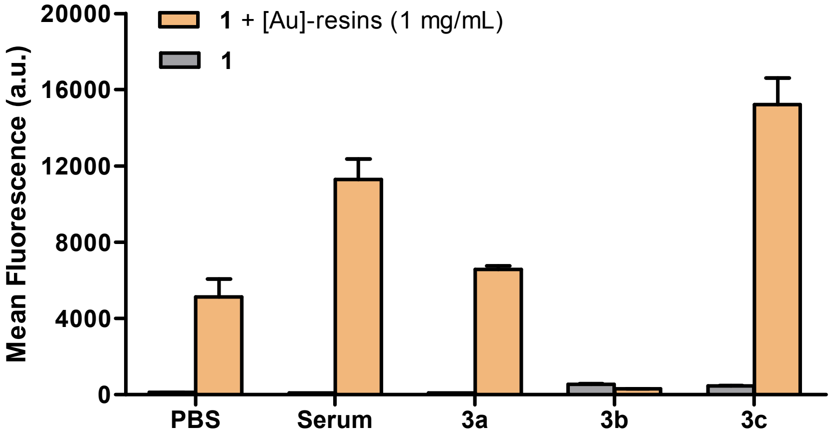

Suppl. Fig. 2. Fluorescence analysis of the reactions of *N*-Poc-protected prodye **1** with or without [Au]-resins after 24 h incubation in the presence of PBS, serum, **3a**, **3b** or **3c** at physiological conditions (pH= 7.4, 37 °C).

Reaction yields were calculated by extrapolation from the standard curve of Rhodamine 110 (**2**) in PBS (below).

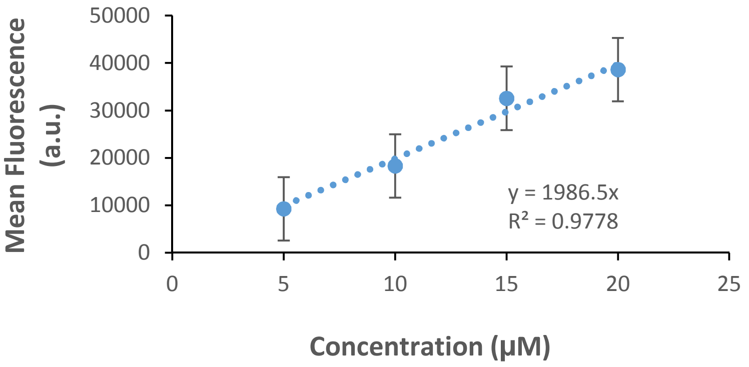

4.2. Reusability study. [Au]-resins (1 mg) were added to a 1 mL solution of compound **1** at 20 μM in PBS or serum (10% in PBS). The mixtures were shaken at 1,200 rpm and 37°C in a Thermomixer and reactions monitored at 24h by fluorescence in a PerkinElmer Victor multilabel reader (excitation filter at 480nm and emissions filter at 535 nm). [Au]-resins (1 mg) were recovered by centrifugation (13,000 rpm, 5 min) and washed with distilled water. A fresh solution of **1** at 20 μM in PBS or serum (10% in PBS) was added to the [Au]-resins, the mixtures shaken at 1,200 rpm and 37 °C, and fluorescence measured at 24 h. This cycle was repeated 5 times.

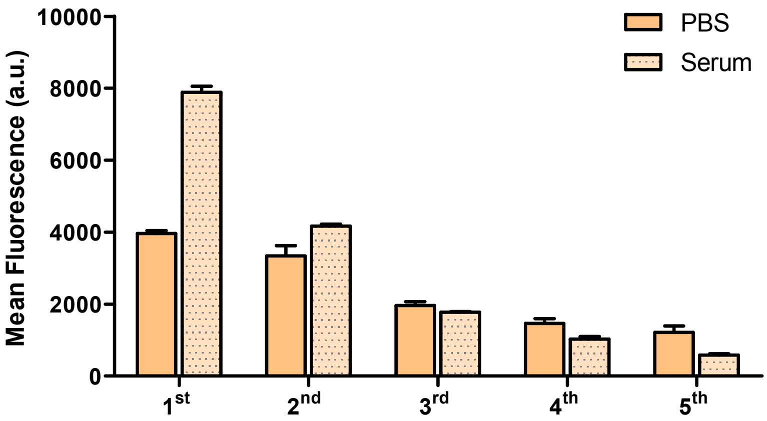

Suppl. Fig. 3. Reusability study. Five successive reactions were performed with 1 mg/mL of [Au]-resins (reused in each cycle) and 20 µM of *N*-Poc-protected prodye **1** in PBS or serum (10% in PBS). A moderate decay in activity after each cycle was observed in all conditions, which could be due to gradual catalyst deactivation, partial loss of Au-NP during the centrifugation steps or to both.

4.3. Time-course study. [Au]-resins (1 mg) were added to a 1 mL solution of compound **1** at 20 µM in serum (10% in PBS). Compound **1** (20 µM) in the absence of [Au]-resins was used as negative control. The mixtures were shaken at 1,200 rpm and 37°C in a Thermomixer and reactions monitored every 24 h by fluorescence in a PerkinElmer Victor multilabel reader (excitation filter at 480nm and emissions filter at 535 nm). A second dose of **1** (1 µL, 20 µM) was added to the [Au]-resins after the measurement on day 3. The experiment was run for 9 d.

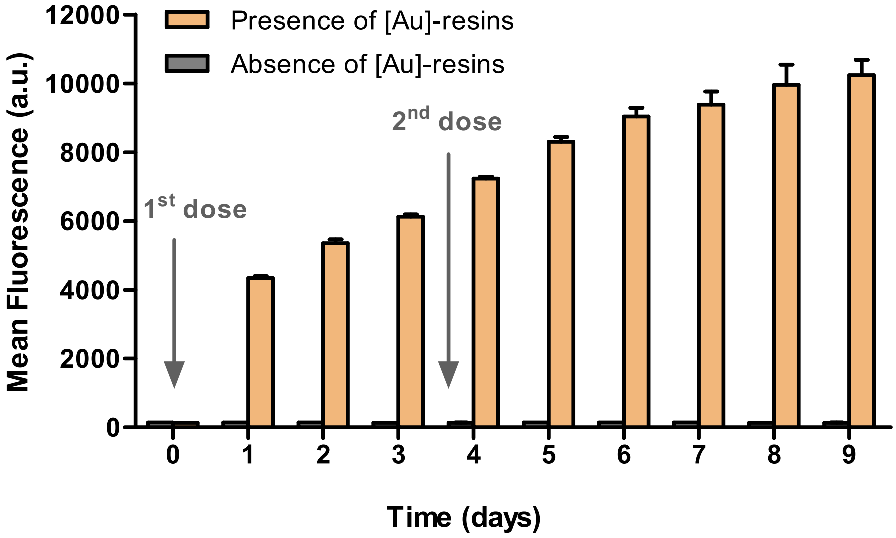

Suppl. Fig. 4. Time-course analysis of fluorescence intensity after incubation of **1** in biocompatible conditions (10% serum in PBS) in the presence (orange) or absence (grey) of [Au]-resins. Reactions were analyzed every 24 h for 9 d. The addition of the doses of **1** is indicated with grey arrows.

4.4. Study of the influence of glutathione. Reactions were performed as above described using PBS as solvent and glutathione as additive at 10 to 400 μM. Fluorescence was measured at 24 h. As shown in Suppl. Fig. 5A, increasing glutathione levels promoted the yield of the reaction compare to the control (no glutathione). However, at ≥ 100 μM, fluorescence levels were inversely proportional to the concentration of glutathione. Next, an extra mg of [Au]-resins was added to the reactions carried out at 100, 200, and 400 μM, and the mixtures were incubated for 24 h. A significant increase of catalytic activity was observed in all samples (see Suppl. Fig. 5B).

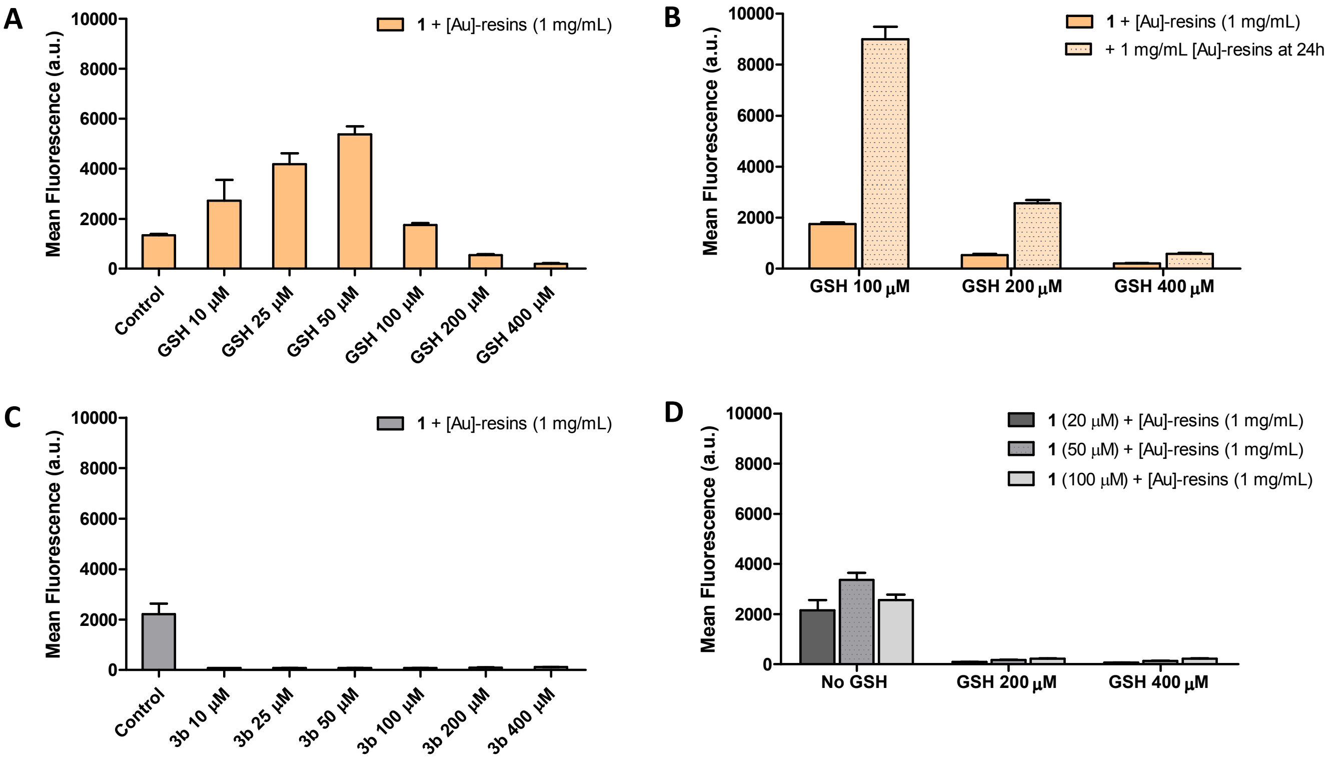

Suppl. Fig. 5. Study of the influence of glutathione (A) or **3b** (C) on the fluorogenic reaction of compound **1** and [Au]-resins. Effect of the increment of the concentration of [Au]-resins (B) or **1** (D) in the reaction. Control: [Au]-resins in PBS without additives.

These results show that glutathione can promote the dealkylation reaction at low-to-medium concentration, most probably via the nucleophilic primary amine present in its structure. To determine whether other functional groups (OH or SH) may assist the reaction in the same way observed with glutathione, reactions were carried out in a range of concentrations of mercaptoethanol (**3b**). Conversely to the effect exhibited by glutathione, compound **3b** inhibited the catalytic activity of the devices at all concentrations (see Suppl. Fig. 5C). The last set of experiments was carried out using probe **1** at higher concentrations (20, 50 and 100 μM) in the presence of inhibitory quantities of glutathione (200 and 400 μM). As shown in Suppl. Fig. 5D, once the glutathione levels have overcome the "inactivation threshold", the reaction is inhibited and the presence of greater concentrations of alkynes do not have a significant effect.

5. Tests performed to identify reaction products and intermediates

5.1. Reaction of 1 with [Au]-resins and benzylamine. TLC-MS analysis. [Au]-resins (45 mg) were added to a solution of **1** (10 mM) and benzylamine (20 mM) in PBS (1 mL). The mixture was shaken at 1,200 rpm and 37°C in a Thermomixer for 72 h. The reaction crude was concentrated under reduced pressure and products separated by semi-preparative TLC (10% MeOH/DCM) before analysis by TLC-MS. Potential alkylated derivatives of benzylamine could not be identified.

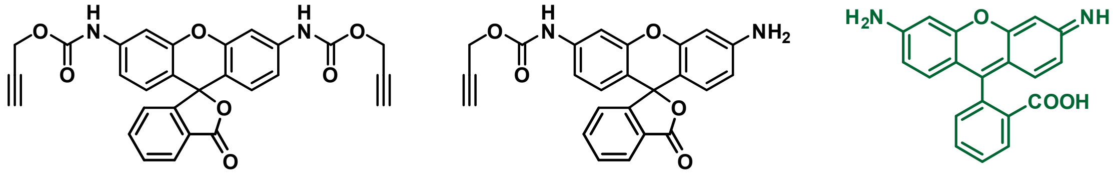

1, MW= 494.46 Mono-Poc Rho, MW= 412.40 **2**, MW= 330.34

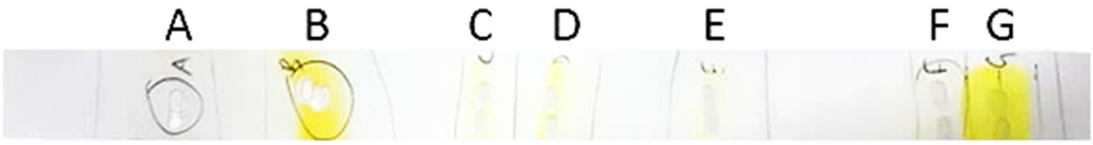

Section of TLC plate (above) showing bands A-G. Bands B, C and G were identified (below).

B) TLC-MS of band B – peak at 495.2845 matches compound **1**

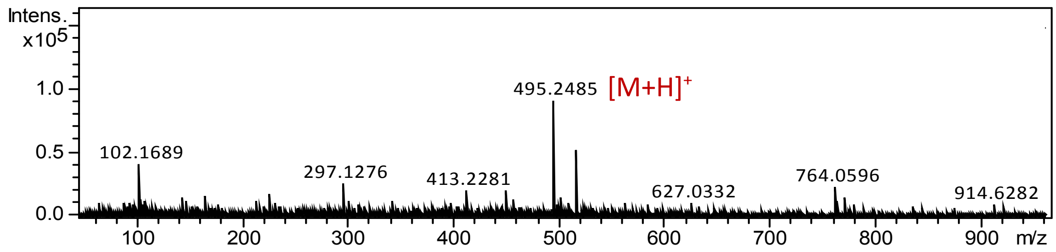

C) TLC-MS of band C – peak at 413.2282 matches mono-protected Poc-Rhodamine

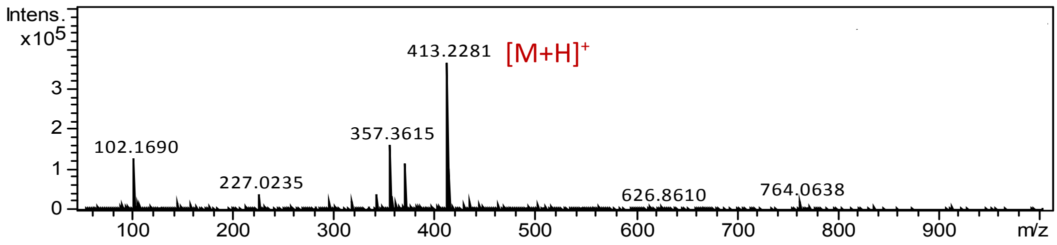

G) TLC-MS of band G – peak at 331.2032 matches Rhodamine 110 (**2**)

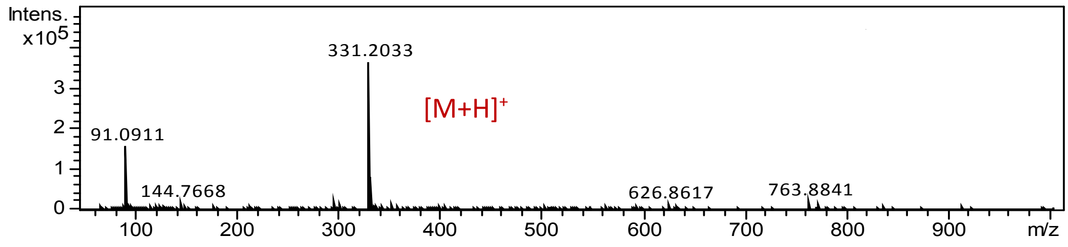

Suppl. Fig. 6. TLC-MS chromatograms of the main bands identified after separating the products of the reaction of **1** with [Au]-resins in PBS.

5.2. Reaction of 6 with [Au]-resins and 3c in DCM. LCMS study. Propargyl *N*-benzylcarbamate (**6**) was synthesised as previously reported [5]. [Au]-resins (10 mg) were added to a solution of **6** (5 mM) and **3c** (15 mM) in DCM (1 mL). This solvent was used to swell the resins and enhance the release of the Au-NP into the solvent to be able to observe organometallic species by LCMS. The mixture was shaken at 1,200 rpm and 37°C in a Thermomixer for 1 h and the reaction crude studied by NMR and LCMS.

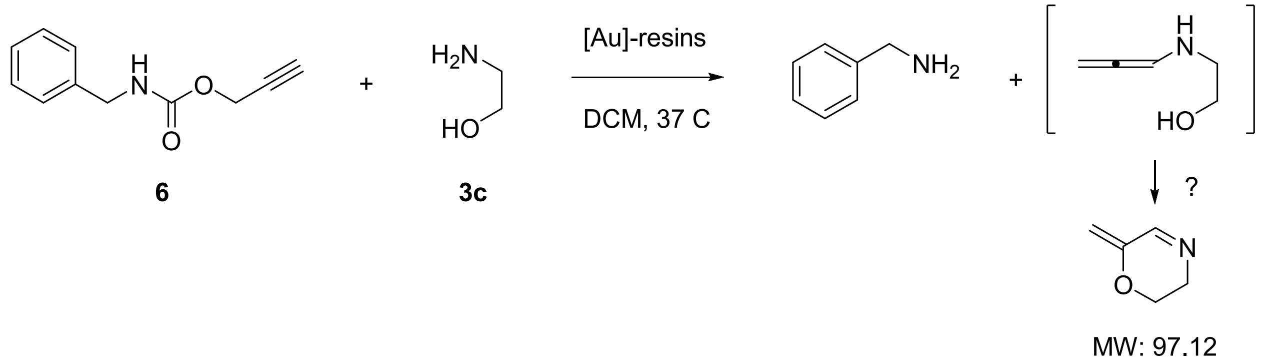

NMR spectra served to identify the formation of benzylamine (1:3 ratio with compound **6**) but other byproducts could not be identified. LCMS analysis showed two peaks that could correspond to organogold species:

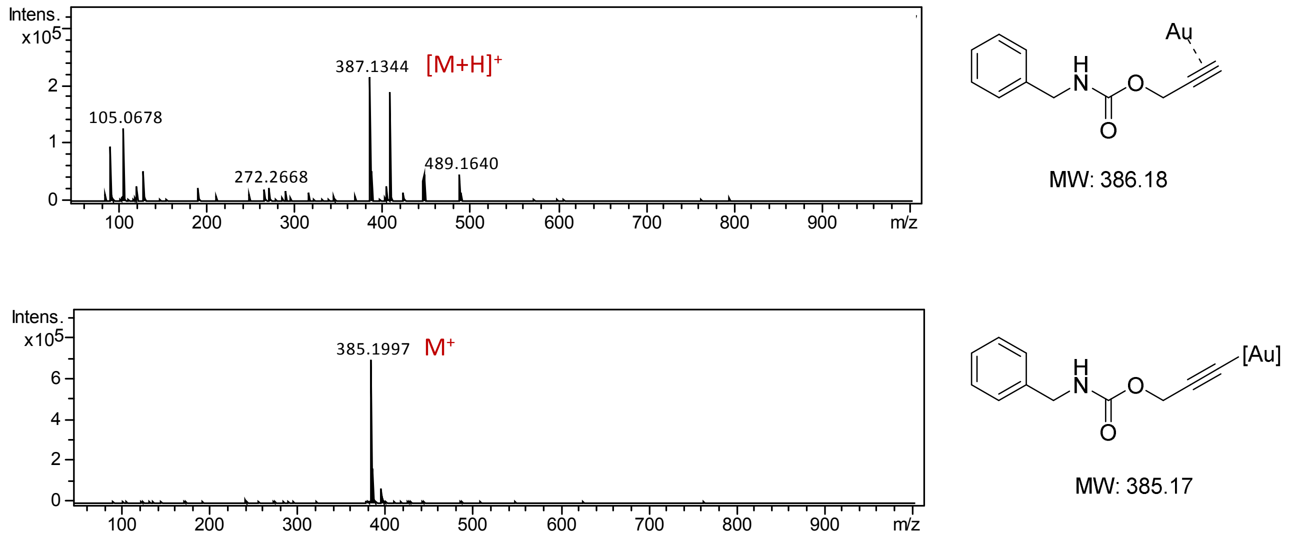

Suppl. Fig. 7. MS chromatograms of two major peaks identified by LCMS after reacting propargyl *N*-benzylcarbamate **6** with [Au]-resins in DCM.

6. Biological studies

6.1. Cell culture. Human lung adenocarcinoma A549 cells (a kind gift from Dr Simon Wilkinson) were cultured in Dulbecco's Modified Eagle Media (DMEM) supplemented with serum (10 % FBS) and L-glutamine (2 mM) and incubated in a tissue culture incubator at 37 °C and 5% CO_2.

6.2. Cell viability studies. Biocompatibility of Au^0-resins was compared by performing dose-response studies in A549 cells. Cells were seeded in a 96-well plate format (at 1,500 cells / well) and incubated for 48h before treatment. Each well was then replaced with fresh media containing [Au]-resins (0.4, 0.6, 0.8, 1 mg/mL) and incubated for 4 d. Untreated cells were incubated with DMSO (0.1 % v/v). Experiments were performed in triplicates. PrestoBlue™ cell viability reagent (10 % v/v) was added to each well and the plate incubated for 90 min. Fluorescence emission was detected using a PerkinElmer Victor multilabel reader (excitation filter at 540nm and emissions filter at 590nm). All conditions were normalized to the untreated cells (100%) and curves fitted using GraphPad Prism using a sigmoidal variable slope curve.

A

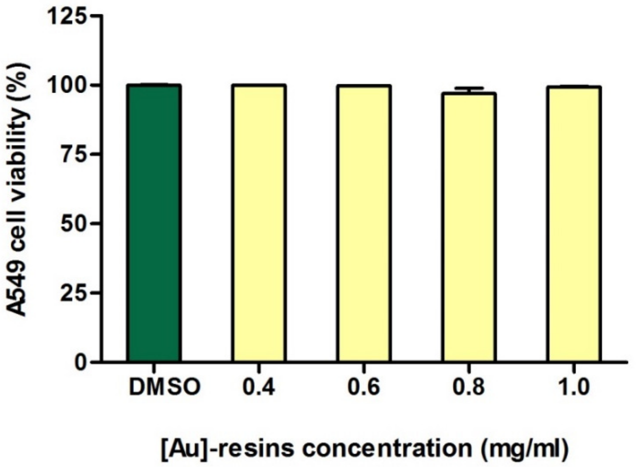

B

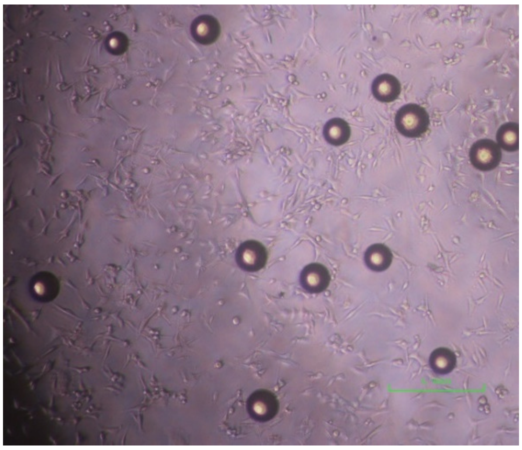

Suppl. Fig. 8. A) Cell viability study of the biocompatibility of [Au]-resins in A549 cells. **B)** Phase-contrast images of U87 cells after 5 days of treatment. [Au]-resins are identified as spheres of approx. 75 µm in average diameter.

6.3. [Au]-mediated activation of 4a-c in cell culture. A549 cells were plated as described above. Each well was then replaced with fresh media containing: [Au]-resins (1mg/mL); **4a-c** (10µM, 100µM and 1µM, respectively); **5a-c** (10µM, 100µM and 1µM, respectively); or combination of [Au]-resins + **4a-c** (10µM, 100µM and 1µM, respectively). All experiments, including the untreated cells, contained 0.1% v/v of DMSO and were performed in triplicates. Cells were incubated with drugs for 4 d. PrestoBlue™ cell viability reagent (10% v/v) was added to each well and the plates were incubated for 90 min. Fluorescence emission was detected and results normalized as described above.

6.4. Multidose prodrug activation study in cancer cell culture. A549 cells were seeded in a 24-well plate format (at 2 x 10^4 cells / well) and incubated for 24 h before treatment. Each well was then replaced with fresh media containing: [Au]-resins (1 mg/mL), **4b** (100μM), **5b** (100μM) or a combination of **4b** and [Au]-resins (1 mg/mL). To facilitate the transferring of the [Au]-resins after each prodrug activation cycle, the [Au]-resins were placed in semipermeable (8 μm pore size) Sarstedt transwell inserts (Suppl. Fig. 9A). Cell viability was determined, as described above, after 2 d of incubation. Before adding the PrestoBlue reagent, the inserts containing the [Au]-resins were moved to a previously seeded 24-well plate and incubated for additional 2 d with fresh media with or without **4b** (100μM). This process was performed twice. All experiments, including the untreated cells, contained 0.1% v/v of DMSO and were performed in triplicates. All conditions were normalized to the untreated cells (100%) as described above (Suppl. Fig. 9B).

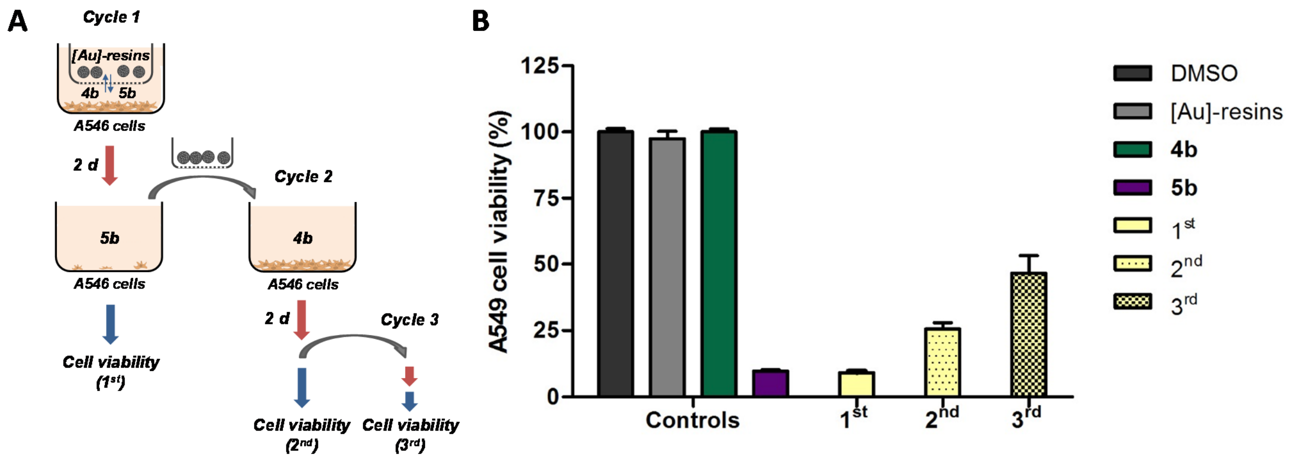

Suppl. Fig. 9. (A) Activation study of multiple prodrug doses in A549 cancer cell culture. Three successive prodrug-to-drug catalytic conversion assays were performed using the same [Au]-resins by transferring the transwell inserts to freshly seeded plates after incubation for 2 d. **(B)** Cell viability analysis of the prodrug activation cycle (labelled as 1st, 2nd and 3rd). Prodrug activation assay: [Au]-resins + **4b** (light yellow). Negative controls: DMSO (untreated cells, dark grey); [Au]-resins (light grey); and prodrug **4b** (green). Positive control: **5b** (purple). Cell viability was measured at day 2 using PrestoBlue reagent. Error bars: ± SD from n = 3.

This study demonstrates the capacity of the [Au]-resins to perform multiple prodrug activation cycles. Nevertheless, the fact that a gradual decay in activity is observed over time suggests some degree of fouling of the catalyst particles by biomolecules, indicating that further development of the device may be required to optimize their durability for clinical use.

7. In vitro prodrug deprotection studies

Prodrugs **4a-c** (200 µM) were dissolved in PBS (500 µL) with 1 mg of [Au]-resins and shaken at 1,200 rpm and 37 °C in a Thermomixer for 4 days. Reaction crudes were dried under a stream of nitrogen and resuspended in 30 µL of methanol. TLC plates were developed in a mixture of DCM/MeOH 9:1 (**4a-b**) and DCM/MeOH 8:2 (**4c**) and imaged under a UV lamp or natural light.

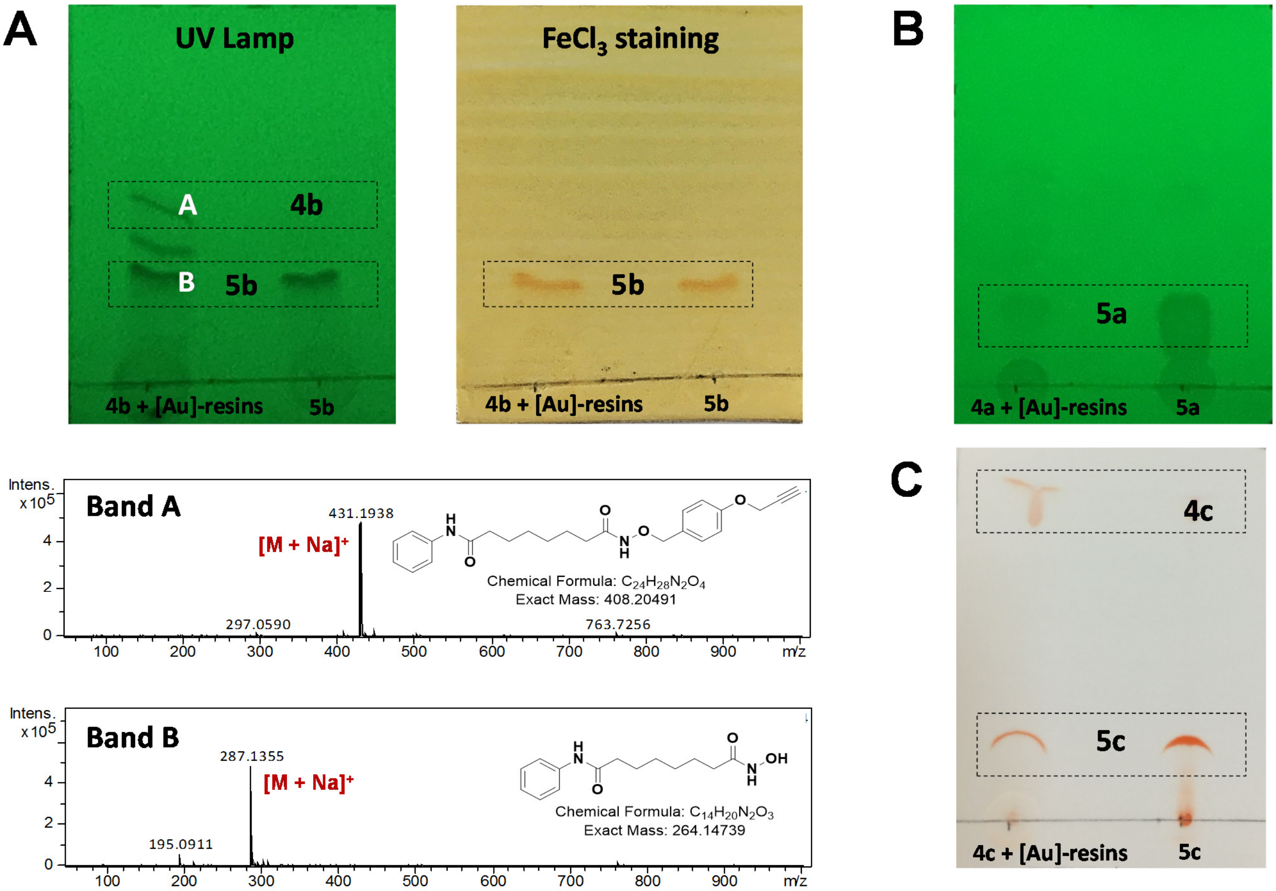

Suppl. Fig. 10. A) TLC images and TLC-MS chromatograms of the reaction of **4b** + [Au]-resins compared to vorinostat **5b**. **B)** TLC image of the reaction of **4a** + [Au]-resins compared to floxuridine **5a** under UV. C) TLC image of the reaction of **4c** + [Au]-resins compared to doxorubicin **5c** under natural light.

Based on the capability of hydroxamic acids to form coloured complexes with ferric ions [4], the TLC plate of the reaction of **4b** and [Au]-resins was also submerged in a $FeCl_3$ solution (60 mM) and dried to differentiate the prodrug **4b** (negative staining) from the resulting drug (stained bands in Suppl. Fig 10A). The spots were further analysed by thin-layer chromatography/mass spectrometry (TLC/MS) using an Advion Nanomate nESI robot.

8. [Au]-resin implantation in zebrafish brain and *gold*-mediated activation of 1

Zebrafish were housed in a purpose build zebrafish facility, in the Queen's Medical Research Institute, maintained by the University of Edinburgh Biological Resources. All zebrafish were kept at 28°C on a 14h light/ 10h dark photoperiod. Embryos were obtained by natural spawning from adult zebrafish. Animal experimentation was approved by the home office, in accordance with the Scientific Procedure Act 1986. Embryos were treated with 1-phenyl 2-thiourea (PTU) during embryogenesis at a concentration of 200 µM to inhibit pigmentation. [Au]-resin implantations were conducted on larvae anaesthetised with 2.5 mM ethyl 3-aminobenzoate methanesulfonate (Tricaine) in zebrafish embryonic medium (6.4 mM KCl, 0.22 mM NaCl, 0.33 mM $CaCl_2$ $2H_2O$, 0.33 mM $MgSO_4$ $7H_2O$), at 3 or 4 days post fertilisation (dpf). Larvae were immobilised during transplantation in 1.5% (w/v) low melting point agarose solution made with zebrafish embryonic medium. Individual implanted zebrafish were maintained in 12-well plates. Implanted zebrafish larvae were then treated with embryo media containing 20 µM of **1** in 1% DMSO and 200µM PTU or only 1% DMSO and 200 µM PTU for a period of 24h. Zebrafish images were obtained using a Zeiss LSM 710 confocal microscope (20 x / 0.8 objective). Image analysis was conducted using Imaris (Bitplane, Zurich, Switzerland) and p values calculated using unpaired student *t*-test.

Time-course assay. The resins were implanted into the brain of the zebrafish at 3 dpf. The experiment comprised four groups: (A) Zebrafish transplanted with [Au]-resin and persistently treated with **1**; (B) zebrafish transplanted with [Au]-resin and intermittently treated with **1**; (C) zebrafish without [Au]-resin and intermittently treated with **1** (negative control); and (D) zebrafish transplanted with [Au]-resin and no treatment (background control). Briefly, the intermittent treatment was: <u>0 to 1 days post transplantation (dpt)</u>, incubation with 20 µM prodye **1** followed by a wash; <u>1 to 3 dpt</u>, incubation in fresh embryo media without prodye; <u>3 to 4 dpt</u>, incubation with 20 µM prodye **1**. The zebrafish were imaged at 1 dpt, 3 dpt and 4 dpt and analysed as described above. Fluorescence intensity in Supp. Fig. 11 is expressed as a ratio to the background control (group 4) for each respective day.

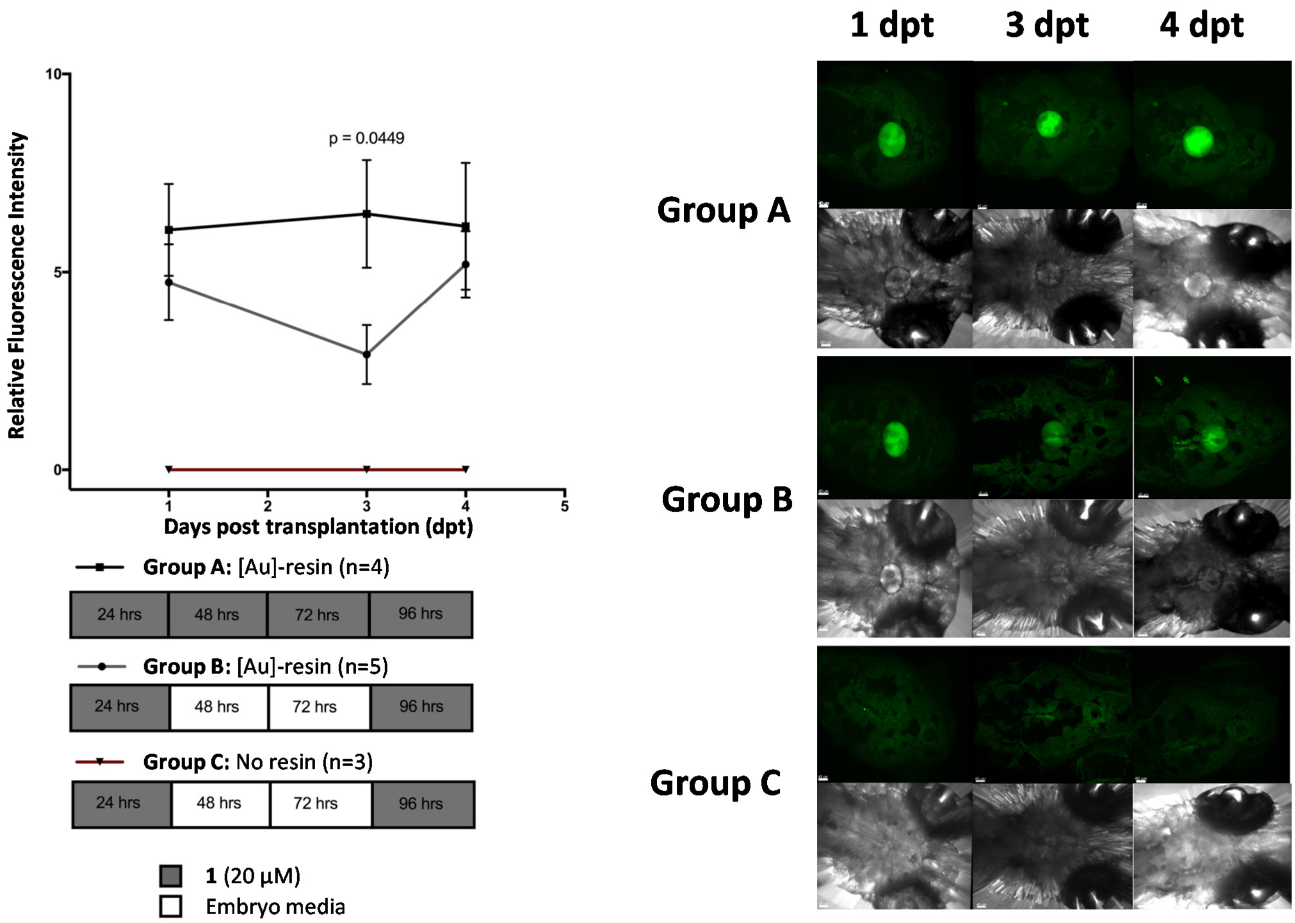

Suppl. Fig. 11. Time-course analysis and representative images of the bioorthogonal gold-mediated release of green fluorescent rhodamine 110 from precursor **1** in the brain of a zebrafish. Study of fluorescence intensity between the persistent (group A, in black) vs the intermittent (group B, in grey) treatment at 3 dpt shows statistical significance.

This study further corroborated previous observations in cancer cell culture. Persistent treatment with reagent **1** (group A), enabled the local generation of dye **2** in the brain and the sustenance of high levels of fluorescence over the life of the study. In contrast, after interrupting the treatment for 2 d (group B), local levels of fluorescence decreased, likely due to the metabolization / elimination of compound **2**. 24 h after resuming the treatment (3 to 4 dpt), high intensity of local fluorescence in the area of the [Au]-resin was restored. Importantly, treatment with reagent **1** in the absence of [Au]-resin did not change fluorescence levels in the brain over the background control.

9. References

[1] A. Corma, H. García, *Chem. Soc. Rev.* **2008**, *37*, 2096-2126.

[2] J. T. Weiss, J. C. Dawson, K. G. Macleod, W. Rybski, C. Fraser, C. Torres-Sánchez, E. E. Patton, M. Bradley, N. O. Carragher, A. Unciti-Broceta, *Nat. Commun.* **2014**, *5*, 3277.

[3] J. T. Weiss, N. O. Carragher, A. Unciti-Broceta, *Sci. Rep.* **2015**, *5*, 9329.

[4] B. Rubio-Ruiz, J. T. Weiss, A. Unciti-Broceta, *J. Med. Chem.* **2016**, *59*, 9974-9980.

[5] R. Ramesh, R. G. Bhat, S. Chandrasekaran. J*. Org. Chem.* **2005**, *70*, 837-840.
